# Effect of a free healthy school meal on fruit, vegetables and unhealthy snacks intake in Norwegian 10- to 12-year-old children

**DOI:** 10.1186/s12889-020-09470-2

**Published:** 2020-09-07

**Authors:** Frøydis N. Vik, Kaia E. P. Heslien, Wendy Van Lippevelde, Nina C. Øverby

**Affiliations:** 1 Department of Nutrition and Public Health, University of Agder, Post-box 422, N-4604 Kristiansand, Norway; 2 Department of Marketing, Innovation and Organisation, Ghent University, Ghent, Belgium

**Keywords:** Free school meal, Fruit, Vegetables, Unhealthy snacks, Intervention, Children, Norway

## Abstract

**Background:**

Norwegian children have a lower intake of fruit, vegetables, and a higher intake of unhealthy snacks compared to dietary guidelines. Such dietary inadequacies may be detrimental for their current and future health. Schools are favorable settings to establish healthy eating practices. Still, no school meal arrangement is provided in Norway, and most children typically bring packed lunches from home. The aim of this study was to investigate whether serving a free healthy school meal for one year resulted in a higher intake of fruit and vegetables and a lower intake of unhealthy snacks in total among 10–12-year-olds in Norway.

**Methods:**

The School Meal Project in Southern Norway was a non-randomized trial in two elementary schools in rural areas in the school year 2014/2015. The study sample consisted of 10- to 12-year-old children; an intervention group (*N* = 55) and a control group (*N* = 109) resulting in a total of 164 school children at baseline. A food frequency questionnaire was completed by the children at baseline, at five months follow-up and after one year to assess fruit, vegetable, and snacks intake. Multiple linear regression analyses were performed to assess intervention effects on overall intake of fruit and vegetables and unhealthy snacks.

**Results:**

Serving of a free healthy school meal for one year was associated with a higher weekly intake of vegetables on sandwiches in the intervention group compared to the control group, adjusted for baseline intake (B: 1.11 (95% CI: .38, 1.85)) at the end of the intervention. No other significant intervention effects were found for the remaining fruit and vegetables measures. Serving of a free healthy school meal was not associated with a lower weekly intake of unhealthy snacks (i.e. potato chips, candy, sugar sweetened beverages) in the intervention group compared to the control group.

**Conclusions:**

A free healthy school meal was associated with a higher weekly intake of vegetables on sandwiches but did not significantly change any other investigated dietary behaviors. However, given the inadequate intake of vegetables among children and that even moderate improvements have public health relevance, a free healthy school meal for all school children could be beneficial.

**Trial registration:**

ISRCTN61703361.

Date of registration: December 3rd, 2018.

Retrospectively registered.

## Background

Dietary imbalances such as low intake of fruit and vegetables (FV) and a high intake of foods high in sugar and saturated fat have been associated with key metabolic/physiologic changes such as overweight/obesity [1], raised blood pressure, blood sugar and cholesterol [2]. Such key metabolic/physiologic changes are risk factors for developing non-communicable diseases (NCD’s) such as cardiovascular disease, diabetes type 2, hypertension, stroke, and cancer which are major global public health threats in the twenty-first century [3, 4]. The link between diet and health in children is also a cause for concern. It is becoming more and more apparent that diet quality early in life affects body composition, physiology and cognition later in life [4]. Moreover, dietary habits are to a large extent established during childhood, and these habits tend to track into adulthood [5–8] underpinning the importance of establishing healthy dietary habits early in life. Also, childhood obesity tracks into adulthood [9]. Recent figures of childhood obesity in Norway from the Child Growth Study show a prevalence of overweight (including obese) of approximately 15% among 8-year-olds [10]. Initiatives that strive to enhance children’s diet are therefore an important part of the Norwegian public health strategy [11].

Schools are important settings for health promotion, since they offer an opportunity to reach almost all children in Norway (96%) from different socio-economic backgrounds [12]. In addition, children spend a considerable amount of time in school and have at least one of their daily main meals there. A meta-analysis showed that school-based interventions can positively impact health behaviours such as dietary habits in primary school children [13]. However, a recent meta-analysis assessed the effectiveness of lunchbox interventions aiming to improve foods consumed by children, and found that the evidence are inconclusive [14]. Evans and colleagues found that school-based interventions moderately improved fruit intake but had minimal impact on vegetable intake [15]. Schools may influence children’s eating habits positively by making healthy foods available and accessible [16].

Currently there is no national arrangement for school meals in Norway, neither free nor parent paid. Consequently, Norwegian school children bring packed lunch to school [17, 18]. Two main concerns are associated with this; some children do not bring lunch and therefore do not eat during school days or eat unhealthy alternatives [19, 20]. Most children in primary school bring a packed lunch, but as they become adolescents, they tend to not bring packed lunch from home [21]. Among the Nordic countries, Denmark has a similar school meal situation as Norway, i.e. packed lunches from home. Sweden and Finland on the other hand serve a free, hot meal every school day to children in primary and secondary schools [22].

The mean daily intake of FV in Norwegian children has been measured in two nation-wide dietary assessments among children in 4th and 8th grade (i.e. in 2000 and 2016) which both showed that approximately only half of the recommended amount of 500 g of FV per day was consumed [23, 24]. Also, the intake of unhealthy snacks was too high in Norwegian children and adolescents [25], despite a decrease in intake of sugar sweetened beverages during the last two decades [26]. Results from a previous Norwegian study serving free school fruit to 6th grade children for 1 year, showed a higher intake of fruits and lower intake of unhealthy snacks 7 years after the intervention was completed [27], while there was not difference at 14 years post intervention [28].

To our knowledge, only one intervention study has previously assessed the effect of serving a free school lunch on dietary habits in Norwegian school children [29]. Ask et al. found that serving of free, healthy school lunch did not lead to an improved intake of fruit, vegetables, low-fat milk, and whole-grain bread, nor reduced intake of unhealthy snacks. A limitation to this study was a short intervention period of 4 months.

There are still challenges concerning children’s diet, and in a public health perspective a free school meal including fruit and vegetables might have the potential to address FV intake and unhealthy snacking behavior [30]. A high intake of fruits and vegetables are important in prevention of chronic diseases [31]. A former Norwegian study found that serving a free piece of fruit at school every day reduced the intake of unhealthy snacks outside school, implicating that the intervention might also influence intake outside school [27]. More research is needed to assess the association between a free healthy school lunch and dietary habits [29]. The aim of the current study was to investigate whether serving of a free school meal (lunch) for 1 year resulted in a higher weekly intake of FV and lower weekly intake of unhealthy snacks in the intervention group compared to the control group after 5 months (during the intervention) and after 1 year (at the end of the intervention).

## Methods

The School Meal Project was a non-randomized trial with an intervention and a control group conducted in the southern part of Norway in the school year 2014/2015, and the current study is part of this project. The study sample consisted of children aged 10 to 12 years from two primary schools. Consumption of FV and unhealthy snacks was assessed with items derived from a validated questionnaire used in the Fruits and Vegetables Make the Marks-project [32]. The school children answered the food frequency questionnaire at school (approximately 45 min) at pre, mid intervention and post intervention. Ethical approval was obtained from the Norwegian Centre for Research Data (NSD) and the ethical committee at the Faculty of Health and Sport Sciences at the University of Agder.

### Sample and procedure

Due to practical considerations, a convenience study sample was used. The cook who prepared the school meal every day lived close to the intervention school, making it feasible to deliver the school meal every day. In order to select another control school, school size and characteristics regarding area (e.g. rural) and location in the same county were considered. Two primary schools participated in the project, and they were both located in a rural area in the same county and were of equal size. One school included both an intervention and a control group, and the other school included a control group. Information about the intervention was given to the principals to get their approval for participation. After this approval was obtained, all children were invited to participate in the study. This constituted a total of 219 invited children. One of the parents/care takers of each child was also invited. Parents were given oral and written information about the study through parent meetings and by an invitation letter. An active written consent of participation regarding their child and themselves was needed in order to participate in the study.

Data were collected at baseline (August/September 2014), in January 2015 (Time 1) and in June 2015 (Time 2). Data from both children and parents were collected at all three data collection points. Parents answered a short questionnaire and only the variable regarding level of education is used in the present study.

### Intervention

The intervention consisted of a free school meal at lunch time served to children in the intervention group for one school year [33]. The school meal was prepared in accordance with current Norwegian dietary guidelines and consisted of whole grain bread (at least 50% whole grain), FV and several types of healthy spread. The spread included butter, fish spread (mostly mackerel in tomato sauce and smoked salmon), cheeses, different kinds of lean meat, liver paste, Norwegian caviar, and eggs. Tomatoes, cucumber, peppers, and lettuce were available to put on the sandwiches, and pieces of fruit and vegetable were served on the side. Natural yoghurt (no added sugar) together with berries were occasionally served. Food high in added sugar and/or saturated fat, like jam and chocolate spread, were not served. No beverages were served as part of the free meal, but children who subscribed to the national milk subscription program received milk, otherwise they were encouraged to drink water. The food was served on trays, and the children helped themselves to the food they preferred. The food was consumed in the classroom, and the children ate together around one or two Tables. A teacher was always present during the meals.

### Measures

#### Personal variables

Parents’ level of education was assessed in the parent questionnaire by two items: “What is your highest level of completed education?” with four response options; “primary school (elementary school or lower secondary school)”, “upper secondary school”, “3-4 years of college or university” and “5 or more years of college or university” and “what is your spouse/partner’s highest level of completed education?”. The response options were the same as the previous item, but also included; “I do not have a spouse/partner”. The parents’ educational level was a proxy for socio-economic status (SES). Both scores were combined and dichotomized into “lower SES” (both parents having completed primary school and upper secondary school) and “higher SES” (at least one parent having completed 3–4 years and more than 5 years of college/university) [34].

#### Intake of fruits and vegetables

Intake of FV was assessed by five food frequency items in the child questionnaire. The items were “How often do you eat vegetables with your dinner?”, “How often do you eat vegetables on your sandwiches?”, “How often do you eat other vegetables (e.g. carrots with your lunch)?”, “How often do you eat apple, orange, pear or banana?” and “How often do you eat other types of fruits or berries (other fruits and berries than apple, orange, pear and banana?”. All questions had ten response options (never, less than once a week, once a week, twice a week, three times a week, four times a week, five times a week, six times a week, every day and several times per day). They were recoded to consumption times per week (0, 0.5, 1, 2, 3, 4, 5, 6, 7 or 10) [27].

#### Intake of unhealthy snacks

The four included unhealthy snack items in this study were assessed by the following questions: “How often do you eat potato chips?”, “How often do you eat candy (e.g., chocolate, mixed candy)”, “How often do you drink fruit squash?” and “How often do you drink soda with sugar?”. All the questions had ten response options (never, less than once a week, once a week, twice a week, three times a week, four times a week, five times a week, six times a week, every day and several times per day). They were recoded to consumption times per week (0, 0.5, 1, 2, 3, 4, 5, 6, 7 or 10). The children were asked to think of their intake during the whole day when they answered these questions.

### Statistical analysis

Preliminary analyses consisting of the descriptive statistics of sample characteristics and key variables were conducted using IBM SPSS 25.0. Descriptive analyses were performed to characterize the sample, and to detect differences between the intervention and the control group. Participants’ characteristics at baseline were compared by independent sample t-tests for quantitative variables and by chi-square tests for qualitative variables to detect baseline differences between the control and the intervention group. Socio-demographics (age, gender and SES) and outcome variables at baseline are presented in Table 1.

**Table 1:** Baseline characteristics for gender, age, parental education as a measure of SES and outcome variables

	Baseline		
	Intervention Mean (SD) or %	Control Mean (SD) or %	***p***-value
**Gender (% girls)**	38	53	.069
**Age (mean, SD)**	11.10 (.32)	11.15 (.92)	.632
**Parental Education (% high)**	53	63	.253
**Vegetables at dinner**	4.55 (2.71)	4.93 (2.52)	.383
**Vegetables on sandwiches**	2.52 (2.51)	2.68 (2.68)	.710
**Other vegetables**	3.62 (2.62)	3.92 (2.60)	.486
**Fruit**	4.52 (2.56)	5.35 (2.75)	.065
**Other fruits**	2.65 (2.32)	3.83 (2.75)	.007*
**Potato chips**	1.37 (1.29)	1.33 (0.98)	.849
**Candy**	1.55 (1.02)	1.46 (1.17)	.634
**Soda with sugar**	1.95 (1.94)	1.64 (1.78)	.337
**Fruit squash**	2.27 (2.42)	1.59 (1.96)	.084

Continuous variables presented with mean (standard deviation)

All outcome variables are continuous on a scale ranging from 0 to 10

*Statistically significant (*p* ≤ 0.05)

The normality of the key variables was checked. Although some of the outcome variables showed a tendency to a skewed distribution, the distribution of the residuals was acceptable. Therefore, the untransformed outcome variables were used. Multiple linear regression analyses were performed to assess intervention effects on the intake of FV and unhealthy snacks (Table 2). All analyses were adjusted for baseline intake. No other covariates were included in the analyses given that they did not significantly associate with the outcomes. All cases with complete data for baseline and follow-ups were included in the analyses.

**Table 2:** Observed means & standard deviations (SD) (times/week) at all three time points and estimated intervention effect (intervention group (IG) vs. control group (CG)) at Time 1 and Time 2 (regression coefficients (Beta (B)) with 95% confidence intervals (CI)) for intake of the various fruit, vegetables and unhealthy snack items (times/week) based on multiple linear regression analyses

**Baseline – Time 1**							**Model 1**		
		**N**		**N**		**N**			
**Variable**			**Mean (SD) BL**^**¥**^		**Mean (SD) Time 1**		**B**	**95% CI**	***p*-value**
**Vegetables at dinner**	**IG**	*55*	4.55 (2.71)	*52*	4.82 (2.35)	*157*	−.34	−1.00, .31	.304
	**CG**	*107*	4.93 (2.52)	*106*	5.31 (2.37)				
**Vegetables on sandwiches**	**IG**	*55*	2.52 (2.51)	*52*	3.12 (2.44)	*155*	.29	−.43, 1.00	.428
	**CG**	*105*	2.68 (2.68)	*106*	2.87 (2.49)				
**Other vegetables**	**IG**	*55*	3.62 (2.62)	*51*	4.14 (2.37)	*156*	−.03	−.84, .77	.936
	**CG**	*107*	3.92 (2.60)	*106*	4.21 (3.10)				
**Fruit**	**IG**	*55*	4.52 (2.56)	*52*	4.84 (2.39)	*156*	−.01	−.78, .76	.984
	**CG**	*106*	5.35 (2.75)	*106*	5.23 (2.75)				
**Other fruits**	**IG**	*55*	2.65 (2.32)	*50*	3.21 (2.35)	*154*	.36	−.43, 1.15	.369
	**CG**	*106*	3.83 (2.75)	*106*	3.50 (2.71)				
**Potato chips**	**IG**	*52*	1.36 (1.29)	*52*	1.33 (1.05)	*152*	.26	−.01, .53	.057
	**CG**	*106*	1.33 (.98)	*106*	1.10 (.76)				
**Candy**	**IG**	*52*	1.55 (1.02)	*52*	1.41 (.93)	*151*	.22	−.01, .45	.062
	**CG**	*105*	1.46 (1.17)	*106*	1.20 (.69)				
**Soda with sugar**	**IG**	*49*	1.95 (1.94)	*51*	1.47 (1.61)	*143*	.04	−.35, .43	.841
	**CG**	*102*	1.64 (1.78)	*103*	1.33 (1.30)				
**Fruit squash**	**IG**	*52*	2.27 (2.42)	*51*	1.75 (2.27)	*148*	.08	−.49, .65	.779
	**CG**	*103*	1.59 (1.96)	*106*	1.40 (1.90)				
**Baseline – Time 2**							**Model 1**		
		**N**		**N**		**N**			
**Variable**			**Mean (SD) BL**^**¥**^		**Mean (SD) Time 2**		**B**	**CI**	***p*-value**
**Vegetables at dinner**	**IG**	*55*	4.55 (2.71)	*52*	4.95 (2.55)	*158*	−.02	−.73, .77	.959
	**CG**	*107*	4.93 (2.52)	*108*	5.10 (2.57)				
**Vegetables on sandwiches**	**IG**	*55*	2.52 (2.51)	*52*	3.68 (2.65)	*155*	1.11	.38, 1.85	.003*
	**CG**	*105*	2.68 (2.68)	*107*	2.63 (2.28)				
**Other vegetables**	**IG**	*55*	3.62 (2.62)	*52*	4.25 (2.56)	*158*	.50	−.31, 1.32	.225
	**CG**	*107*	3.92 (2.60)	*108*	3.79 (2.68)				
**Fruit**	**IG**	*55*	4.52 (2.56)	*52*	4.94 (2.59)	*157*	.18	−.62, .98	.656
	**CG**	*106*	5.35 (2.75)	*108*	5.11 (2.71)				
**Other fruits**	**IG**	*55*	2.62 (2.32)	*52*	3.15 (2.12)	*157*	.33	−.38, 1.04	.363
	**CG**	*106*	3.83 (2.75)	*108*	3.04 (2.16)				
**Potato chips**	**IG**	*52*	1.36 (1.29)	*52*	1.08 (.86)	*153*	.05	−.17, .27	.648
	**CG**	*106*	1.33 (.98)	*107*	1.04 (.85)				
**Candy**	**IG**	*52*	1.55 (1.02)	*52*	1.38 (1.39)	*151*	.24	−.08, .55	.135
	**CG**	*105*	1.46 (1.17)	*106*	1.18 (.72)				
**Soda with sugar**	**IG**	*49*	1.95 (1.94)	*52*	1.57 (1.79)	*145*	.36	−.05, .77	.084
	**CG**	*102*	1.64 (1.78)	*105*	1.07 (1.02)				
**Fruit squash**	**IG**	*52*	2.27 (2.42)	*51*	1.94 (2.31)	*149*	.21	−.39, .81	.498
	**CG**	*103*	1.59 (1.96)	*107*	1.59 (2.05)				

¥: BL = baseline

Model 1: adjusted for baseline intake

Time 1 = 5 months into the intervention and Time 2 = at the end of the intervention

*Statistically significant (*p* ≤ 0.05)

## Results

### Study characteristics

Of the 219 invited children, 168 received an active written consent from a parent, but four of them chose not to participate. Thus, the baseline study sample consisted of 164 children (participation rate 75%). The intervention group consisted of 55 children from 6th grade, while the control group consisted of 109 children from 5th, 6th, and 7th grade. This gave a 96% participation rate in the intervention group and a 67% participation rate in the control group at baseline. A flow chart of the children’s participation and those lost to follow up has been published elsewhere [33].

At baseline, there were 55 participants in the intervention group and 109 participants in the control group. In the intervention group, there were 38% girls and 53% with higher SES. In the control group, there were 53% girls and 63% with higher SES. The mean age at baseline was 11.10 ± .32 years in the intervention group, and 11.15 ± .92 years in the control group. There were no statistically significant differences between the intervention and control group with regards to gender, age or SES and outcome items at baseline, apart from “intake of other fruits” (i.e. other fruits or berries than apple, pear, banana or orange), in favour of the control group (*p* = .007) (Table 1).

### Associations of the intervention on FV and unhealthy snack intake

The multiple linear regression analyses showed that the intervention was positively associated with the mean frequency intake of vegetables on sandwiches compared to the control group at follow-up 2 after adjusting for baseline intake (B: 1.11 (95% CI: .38, 1.85) (Table 2). However, the analyses did not show any other statistically significant associations with frequency of intake of vegetables at dinner, other vegetables (e.g. carrots with lunch), fruit (apple, orange, pear or banana) or other fruit (other fruits or berries than apple, orange, pear and banana) in the intervention group compared to the control group (Table 2). Serving of a free healthy school meal was not associated with a lower frequency of intake of unhealthy snacks (potato chips, candy, fruit squash and soda) in the intervention group compared to the control group after adjusting for baseline intake (Table 2).

## Discussion

Results from this study showed that serving of a free, healthy school meal for 1 year was significantly associated with weekly frequency of intake of vegetables on sandwiches in the intervention group compared to the control group at the end of the intervention but not in the middle of the intervention. The intervention did not show other statistically significant associations in the intervention group compared to the control group with regards to frequency if intake of other vegetables, fruits, or unhealthy snacks.

The increased frequency of intake of vegetables on sandwiches in the intervention group is important because bread is an essential staple of the Norwegian every-day diet. Children, as do adults, have a high frequency of consumption of bread based meals, with 82, 85 and 61% consumption for breakfast, lunch and supper, respectively [35]. Since the intake of bread is so high, the use of vegetables on sandwiches might be a potential way to increase vegetable intake, which we know is lower than the dietary recommendations [25]. Since vegetables commonly used for sandwiches (lettuce, tomatoes, cucumber, peppers) were part of the free school meal, it is likely that the significantly increased intake of vegetables on sandwiches in the intervention group may be due to what the children ate at school. However, it is also possible that the increased frequency of intake may be explained by an increased consumption at home and during leisure time. This could potentially be explained by habit theory, in that repeatedly performing a behaviour, i.e., intake of vegetables on sandwiches at school every day for 1 year, might result in it becoming a habit [36, 37]. We did not observe this effect at Time 1, and this might be explained by the fact that the intervention may have changed the home environment, but that it took time to do so. Finally, it is important to emphasize that although a significant increase in frequency was detected, it was minimal.

The average frequency of intake of unhealthy snacks among the participating children was relatively low at both baseline and the follow-up assessments. The lack of associations by the intervention might therefore be due to little possibility for improvement with regards to the frequency of intake of unhealthy snacks, as it was already low at baseline. Also, the intervention did not target intake of snacks specifically, as it was a free healthy school meal intervention, and this may be an explanation why we did not notice any change. There has been a clear decrease in sugar intake among Norwegian children since the turn of the millennium [23, 24]. The lack of associations by the intervention might therefore be due to a general decrease in sugar intake in the Norwegian child population, and not the intervention itself, and might also explain the difference in results from this study compared to the free school fruit study [27], which was done early 2000.

The former study by Ask et al. found that a free school lunch for 4 months did not improve the intake of fruit, vegetables, low-fat milk and wholegrain bread, or reduce the intake of unhealthy snacks [29]. The yearlong intervention period in the current study posits a larger potential for intervention effect as it gives more time to detect a possible effect. This might explain why the current study had a positive effect on vegetable intake, while the study conducted by Ask, et al. did not lead to any improved measures on diet.

This study might be promising, but the associations were small. Further studies to measure the actual intake of fruit and vegetables to evaluate the true impact on vegetable intake is needed. If provision of fruit and vegetables at school is combined with nutrition education, it may be more effective [38].

### Strengths and limitations of the study

There are several strengths and limitations to this study. Strengths are the long intervention period of 1 year, a high participation rate, and having a control group and an intervention group including baseline comparisons. It is also a strength that the study has three data collection points, which makes it possible to assess how the outcome of the intervention develops over time. The same research assistants conducted the data collection at all timepoints. The questionnaire was pilot tested in the same age group before the study started.

Limitations to this study are non-randomization, small sample size and self-reported data. Given that the study was non-randomized, causality is limited. We have therefore used associations to describe the results, instead of intervention effect. Potential biases are likely to be higher for non-randomized studies compared with randomized trials when evaluating the effects of interventions [39], so our results should be interpreted with caution. The control group was selected based on similarities to the intervention group, making it reasonable to assume that possible differences are very limited. Analyses conducted also revealed that the two groups generally did not differ significantly. The fact that the intervention group was located at the same school as part of the control groups represents a substantial limitation. However, the children in the intervention group were in a totally different part of the school building than the control group, minimizing the chance of bias. They also ate the school meal in the classroom, and not in a school canteen, making it less visible to the control children. Minor differences in age as well as differences in group size between the intervention group and the control group constitute another limitation. Large sample sizes are preferred in quantitative research, but the nature of this intervention made a larger sample unrealistic. The FFQ used in this study was quite limited, both regarding the lack of details in fruits and vegetables eaten and the lack of portion sizes since only frequency was measured, and a better dietary method would have strengthened the study. Self-reported data may be biased if respondents do not fully remember what they have eaten, or if they over/under-report their intake [40]. Regarding self-reported data, we know from other fields that children in this age group are capable of answering items regarding themselves [41, 42]. Children in the School Meal Project were aware that improved diet quality was a desired outcome. This might have made them report healthier habits than they really have, which is a common phenomenon known as “social desirability bias” [43]. These data were collected from school children in rural areas. We cannot conclude that these results would be the same for urban areas, as we do not have national data in Norway that support this. The most recent report (2015) on dietary intake among Norwegian 4th graders and 9th graders state that there are marginal differences among different regions of the country, but differences in urban/rural areas are not included [23].

## Conclusions

A free healthy school meal was associated with a higher weekly frequency of intake of vegetables but was not associated with a significant change in any if the other dietary behaviors investigated. However, given the inadequate intake of vegetables among children it may represent an opportunity to increase intake of vegetables among children in Norway if a free healthy school meal for all school children is provided. Free school meals may have a potential for health promotion and on improving future public health measures among children.

## Data Availability

The datasets used and/or analysed during the current study are available from the corresponding author on reasonable request.

## Abbreviations

FV: Fruit and vegetables
NCD’s: Non-communicable diseases
NSD: Norwegian centre for research data
SES: Socio-economic status
BL: Baseline
